# The Trouble with Sliding Windows and the Selective Pressure in BRCA1

**DOI:** 10.1371/journal.pone.0003746

**Published:** 2008-11-18

**Authors:** Karl Schmid, Ziheng Yang

**Affiliations:** 1 Department of Plant Biology and Forest Genetics, Swedish University of Agricultural Sciences (SLU), Uppsala, Sweden; 2 Department of Biology, University College London, London, United States of America; Centre for Genomic Regulation, Spain

## Abstract

Sliding-window analysis has widely been used to uncover synonymous (silent, *d_S_*) and nonsynonymous (replacement, *d_N_*) rate variation along the protein sequence and to detect regions of a protein under selective constraint (indicated by *d_N_*<*d_S_*) or positive selection (indicated by *d_N_*>*d_S_*). The approach compares two or more protein-coding genes and plots estimates *dˆ* *_S_* and *dˆ* *_N_* from each sliding window along the sequence. Here we demonstrate that the approach produces artifactual trends of synonymous and nonsynonymous rate variation, with greater variation in *dˆ* *_S_* than in *dˆ* *_N_*. Such trends are generated even if the true *d_S_* and *d_N_* are constant along the whole protein and different codons are evolving independently. Many published tests of negative and positive selection using sliding windows that we have examined appear to be invalid because they fail to correct for multiple testing. Instead, likelihood ratio tests provide a more rigorous framework for detecting signals of natural selection affecting protein evolution. We demonstrate that a previous finding that a particular region of the BRCA1 gene experienced a synonymous rate reduction driven by purifying selection is likely an artifact of the sliding window analysis. We evaluate various sliding-window analyses in molecular evolution, population genetics, and comparative genomics, and argue that the approach is not generally valid if it is not known *a priori* that a trend exists and if no correction for multiple testing is applied.

## Introduction

Sliding-window analysis is a popular graphical method for visually revealing trends in synonymous and nonsynonymous rate variation along a protein sequence, and for identifying protein regions that are under functional constraint or positive selection [e.g.], [ 1], [2]–[5]. It is implemented in several computer programs and web servers [e.g.6 ], [7], [8]. Because of its simplicity and intuitive appeal, its legitimacy in such analyses was most often taken for granted.

When applying the approach to compare various gene sequences, we noted two features of the analysis: (i) the estimated number of synonymous substitutions per synonymous site (*dˆ* *_S_*) and the number of nonsynonymous substitutions per nonsynonymous site (*dˆ* *_N_*) always showed clear trends along the protein sequence, and (ii) *dˆ* *_S_* was more variable than *dˆ* *_N_* along the gene sequence. The greater variation of *dˆ* *_S_* than of *dˆ* *_N_* is particularly surprising. Because processes operating at the DNA level, such as local mutation rate variation [9], should affect both *d_S_* and *d_N_* [10: p. 65] while natural selection on the protein should affects *d_N_* but not *d_S_*, and because protein-level selection is expected to vary across amino acid sites or protein domains, we expect *d_N_* to be more variable than *d_S_* [see also 3]. For *d_N_* to be less variable than *d_S_*, variation in selective constraint on the protein will have to counterbalance variation in mutation rate. Such a scenario appears to be too contrived to apply to many genes. Further examinations, however, suggest that the apparent trends in *dˆ* *_S_* and *dˆ* *_N_* revealed by sliding-window analysis do not reflect variations in the true *d_S_* and *d_N_*, and are an artifact of the procedure. The effect is inherent in the method and affects many applications of sliding-window analysis.

Here we demonstrate the artifactual effect of sliding-window analysis through a re-analysis of the breast-cancer gene BRCA1 from mammalian species. We also discusses similar problems when sliding-window analysis is used in several other applications in molecular evolutionary studies.

## Results

### Sliding-window analysis of mammalian BRCA1 genes

The breast-cancer gene BRCA1 is a well-known empirical case of synonymous rate variation, since Hurst and Pál [3], [9] conducted a sliding-window analysis to compare the human with the dog and the mouse with the rat genes. Here we reanalyze the data to show that the apparent synonymous rate variation and the purifying selection acting on silent sites in a particular region inferred by those authors is likely an artifact. We follow the common practice of conducting sliding-window analysis in pairwise sequence comparisons but note that our conclusions apply also to simultaneous comparison of multiple sequences. Besides the mouse-rat and human-dog pairs, we also use the orangutan-cow and orangutan-macaque pairs.

**Figure 1:**
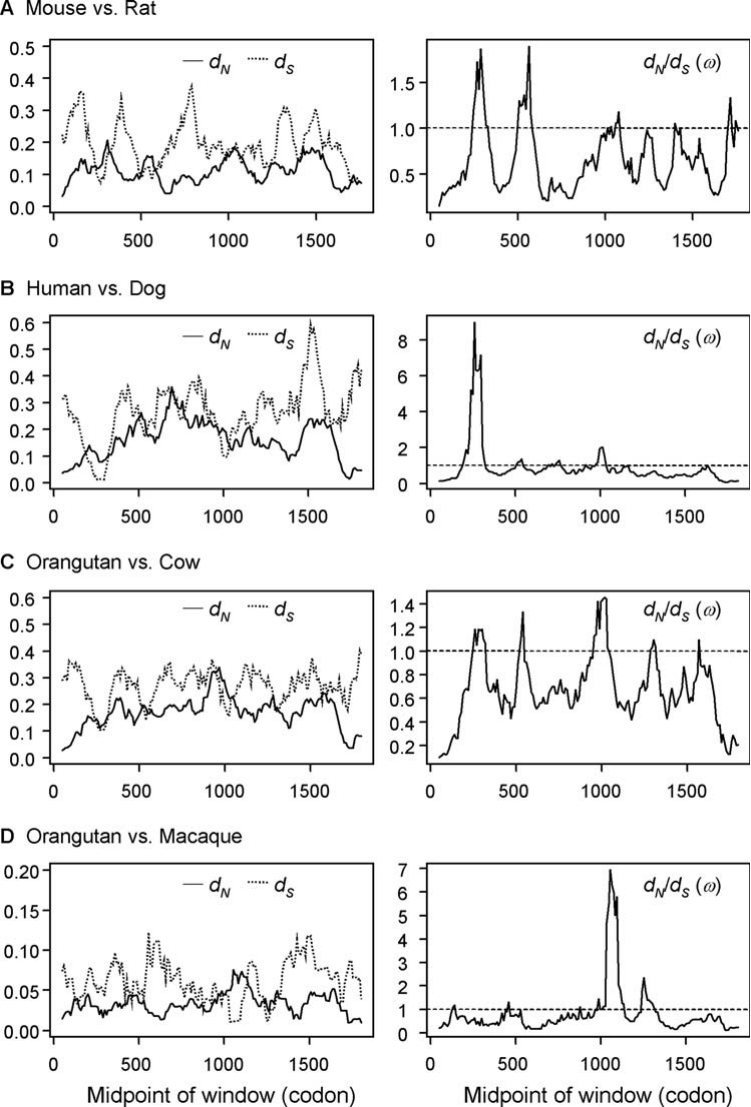
Sliding-window plots of *dˆ* *_S_*, *dˆ* *_N_* and *ωˆ* = *dˆ* *_N_*/*dˆ* *_S_* in pairwise comparisons of the BRCA1 genes from mammalian species. The window size is 100 codons, and the offset between windows is one codon.

The results are presented in Figure 1. The window size is set to 100 codons, with an offset of one codon between successive windows. In each window, the *ω* ratio ( = *d_N_*/*d_S_*) as well as *d_S_* and *d_N_* were estimated using maximum likelihood (ML) under model M0 (one-ratio), which assumes that the same *ω* ratio applies to all codons in the gene [11]. While the method for estimating *d_S_* and *d_N_* may be important, the effects we demonstrate do not depend on the estimation method; use of the approximate methods such as YN00 [12] produced qualitatively identical results (not shown). From Figure 1, the following patterns are apparent: (i) both *dˆ* *_S_* and *dˆ* *_N_* show smooth trends of fluctuation along the sequence; (ii) *dˆ* *_S_* fluctuates more wildly along the sequence than *dˆ* *_N_*; and (iii) in some regions, the estimated rate ratio *ωˆ*>1, which could naïvely be interpreted as indicating positive selection.

As discussed by Hurst and Pál [3], there is a striking plummet in *dˆ* *_S_* around codon 250 in the comparisons between the mouse and the rat and between the human and the dog (Figure 1A&B). Hurst and Pál referred to this region as the ‘critical region’ and their test suggested that the *ω* ratio was significantly greater than 1 in the human-dog pair and significantly higher than the average for the whole gene in the mouse-rat pair. The authors suggested purifying selection at silent sites as the most likely mechanism for the reduced *dˆ* *_S_* and for the elevated *ωˆ* for the region. Nevertheless, the authors' tests do not appear to be valid, because the ‘critical region’ was identified by analyzing the data and not specified *a priori*, and because no correction for multiple testing was applied (see below). The orangutan-macaque comparison (Figure 1D) is largely independent phylogenetically of the mouse-rat and human-dog comparisons, and does not show a dip in *dˆ* *_S_* in the critical region. The orangutan-cow comparison (Figure 1C) overlaps somewhat with the human-dog comparison, and shows a small dip in *dˆ* *_S_* in the critical region, but is by no means out of the ordinary. It is noteworthy that even between the mouse-rat and human-dog comparisons, the peaks and valleys in *dˆ* *_S_* and *dˆ* *_N_* do not occur at similar locations except for the dip in *dˆ* *_S_* in the critical region.

### Sliding-window analysis of simulated data

**Figure 2:**
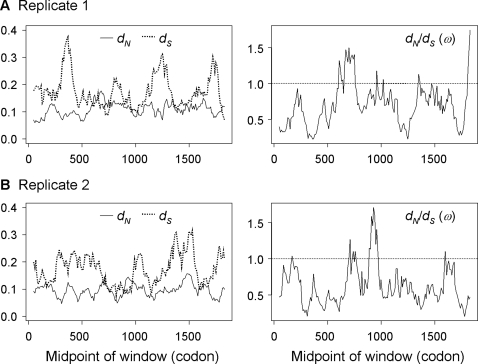
Sliding window plots of *dˆ* *_S_*, *dˆ* *_N_* and *ωˆ* = *dˆ* *_N_*/*dˆ* *_S_* from two simulated data sets, generated under model M0 (one-ratio) using parameter estimates obtained from the comparison of the mouse and rat BRCA1 genes. The window size is 100 codons, and the offset between windows is one codon.

To examine whether the patterns of Figure 1 are statistically significant and may thus reflect real biological processes, we apply the sliding-window analysis to data sets simulated under model M0 (one-ratio), which assumes the same *d_S_*, *d_N_*, and *ω* across the whole sequence and independent evolution among codons. The ML estimates of parameters under M0 from the original pair of real sequences [11] were used to simulate replicate data sets using program evolver in the paml package [13]. The results obtained from simulations based on the four pairs of sequences are qualitatively similar, so we present in Figure 2 only those for the first two replicate data sets based on the mouse-rat comparison. The original parameter estimates for this pair are *tˆ* = 0.391, *κˆ* = 3.304, and *ωˆ* = 0.504, with *dˆ* *_S_* = 0.204 and *dˆ* *_N_* = 0.103.

Simply from visual inspection, we were unable to distinguish the plots in Figure 1A for the real data from those in Figure 2A&B for the simulated data. The peaks and valleys in *dˆ* *_S_* and *dˆ* *_N_* in Figure 2 are random and differ between simulated replicates. However, like the real data, the simulated data show considerable and smooth fluctuations in *dˆ* *_S_* and *dˆ* *_N_*, greater fluctuations in *dˆ* *_S_* than in *dˆ* *_N_*, and also windows with *ωˆ*>1. All those features are artifactual.

We suggest that the following reasons may explain the features. First, *dˆ* *_S_* and *dˆ* *_N_* calculated from the sliding windows will fluctuate due to chance effects in a small window. Because two neighboring windows share many codons, *dˆ* *_S_* and *dˆ* *_N_* will change smoothly when plotted against the sequence. Of course the amount of smoothing depends on the window size and the offset between consecutive windows. Second, the fluctuations in *dˆ* *_S_* and *dˆ* *_N_* are due to fluctuations in the estimated numbers of synonymous (*S_d_*) and nonsynonymous (*N_d_*) substitutions and in the numbers of synonymous (*S*) and nonsynonymous (*N*) sites. Consider the numbers of sites *S* and *N* in a window. Their sum is 3*w*, where *w* is the number of codons in each window. Random fluctuations in amino acid composition or codon usage will generate fluctuations in *S* and *N*. Because *N* is about three times as large as *S*, the same amount of change will proportionally affect *S* much more than it affects *N*. As a result, *dˆ* *_S_* tends to fluctuate more than *dˆ* *_N_*. Because the data of Figure 2 are generated under model M0 (one-ratio), with constant *d_S_* and *d_N_* along the sequence and with independent evolution among codons, the apparent variation and trends in *dˆ* *_S_* and *dˆ* *_N_* are artifacts.

### Multiple testing in sliding-window analysis and likelihood ratio test of positive selection

We examined the validity of previous uses of sliding-window analysis to test for regions of a protein under selective constraint or positive selection [e.g.], [1], [4], [7], [8,14]. Most such studies used simplistic methods to estimate *d_S_* and *d_N_*, ignoring major features of DNA sequence evolution such as unequal codon frequencies or different transition and transversion rates. Here we claim that most such tests we have examined appear to be invalid, partly because they did not correct for multiple testing. If one conducts 100 independent tests at the 5% significance level, one is expected on average to reject falsely the null hypothesis by chance in 5 tests. Here the tests are not independent because the windows overlap, but the problem of multiple testing exists. The overall false-positive rate or the family-wise error rate refers to rejection of at least one true null hypothesis when multiple null hypotheses are tested. This error rate can be much higher than the significance level if no correction for multiple testing is applied.

**Figure 3:**
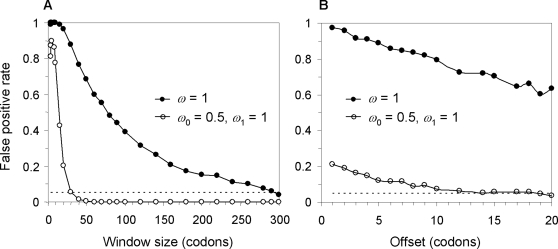
The overall false-positive rate of the sliding-window test of positive selection plotted against the window size. Data of a pair of sequences are simulated under either model M0 (one-ratio) with *ω* = 1 (•) or model M1a (neutral) with two site classes in proportions *p*_0_ = *p*_1_ = ½ with *ω*_0_ = 0 and *ω*_1_ = 1 (○). An LRT is used to test for positive selection in each window, by fitting model M0 (one-ratio) to the data, either with *ω*≥1 estimated or with *ω* = 1 fixed, and by comparing twice the log likelihood difference between the two hypotheses with 2.71, at the 5% level. If the test is significant in any window, positive selection is claimed to be detected for the replicate data set. The false-positive rate is calculated as the proportion of replicate datasets in which the test is significant in at least one window. The sequence length is 300 codons. The impact of the window size is examined in A, with the offset fixed at one codon, while the impact of the offset is examined in B, with the window size fixed at 20.

Figure 3A shows the relationship between the overall false-positive rate and the size of the sliding window, when a pair of sequences is simulated under a model of no positive selection and then analyzed using sliding windows to test for positive selection. We used two null models to simulate the data. The first is model M0 (one-ratio) with the single *ω* ratio fixed at 1. The second is the site model M1a (neutral), which assumes two site classes with *ω*_0_ = 0 and *ω*_1_ = 1, in proportions *p*_0_ = *p*_1_ = ½. Each simulated data set is analyzed using a sliding window, using an LRT to test whether *ωˆ* for that window is significantly greater than 1. A false positive is recorded if the test is significant in at least one window. The error rate rises quickly with the increase of the window size, peaks at an intermediate window size of between 5 and 10 codons, and then drops with the further increase of the window size. The false positive rates are unacceptably high at low and intermediate window sizes. Note that in datasets simulated under M1a, the overall error rate is nearly zero in large windows, because the test based on M0 (one-ratio), which requires the average *ω* ratio for the whole sequence to be >1, is very stringent. The effect of the offset is examined in Figure 3B, which shows that for a fixed window size (20 codons), the error rate drops when the offset increases.

A simulation approach may be used to correct for multiple testing. One may use the number of windows in which *ωˆ*>1 as the test statistic; let this be *W*. The null distribution can be generated by simulating under a null model of no positive selection. An appropriate null model is the site model M1a (neutral), which assumes two site classes with *ω*_0_<1 and *ω*_1_ = 1 [15]. We applied this test to the four pairs of BRCA1 genes. For each pair, we calculated the test statistic from the original data, *W*. The original data were then used to estimate parameters under M1a (neutral), and the estimates were used to simulate 1000 datasets under M1a. Each dataset *i* was then analyzed using a sliding window to calculate the number of windows in which *ωˆ* under M0 is >1, *W* ^(*i*)^. The *p* value is the proportion of simulated datasets in which *W* ^(*i*)^≥*W*. We used the window size of 100 codons, with an offset of 10 codons to analyze the BRCA1 genes. The results are shown in table 1. The test is significant in the human-dog (*p*<1%) and orangutan-cow (*p*<5%) pairs, but not in the mouse-rat and orangutan-macaque pairs.

**Table 1:** Test of sites under positive selection by the sliding-windows analysis and by the LRT.

Data	Sliding windows		2Δℓ	2Δℓ
	Statistic a	*p*-value	(M1a-M2a)	(M7-M8)
Mouse-rat	19	0.096	2.24	2.39
Human-dog	18	0.007**	8.18*	9.08*
Orangutan-cow	23	0.016*	0.35	0.40
Orangutan-macaque	16	0.762	0.00	0.00

a The test statistic in the sliding window analysis is the number of windows in which *ωˆ*>1. The window size is 100 codons, and the offset is 10 codons. In the LRTs, the test statistic 2Δℓ is the log likelihood difference between the null and alternative models.

* : significance with *p*<5%.

** : significance with *p*<1%.

For comparison, we applied two likelihood ratio tests of positive selection to the same data, comparing the site models M1a (neutral) against M2a (selection) and M7 (beta) against M8 (beta&*ω*). Both LRTs are significant in the human-dog comparison and not significant in all other pairs (table 1). We note that the test based on sliding windows is a goodness of fit test, although the test statistic is designed such that rejection of the null indicates positive selection.

**Figure 4:**
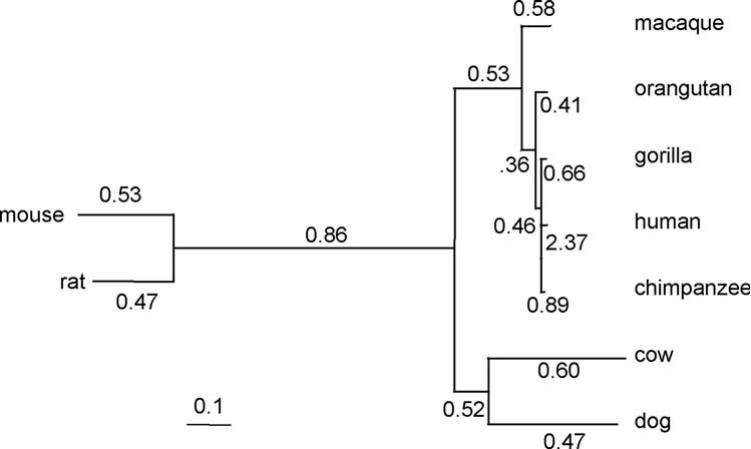
The phylogeny for nine mammalian species. The branch lengths, in the expected number of nucleotide substitutions per codon, are estimated under the free-ratios model [18] from analysis of the BRCA1 genes, while the estimated *ω* ratios for branches are shown along the branches.

Previous simulation studies suggest that the LRTs based on site models may be more sensitive when multiple sequences are compared jointly on a phylogenetic tree [16], so we applied the LRTs to the dataset of nine mammalian species. The phylogeny is shown in Figure 4. The results are summarized in table 2. M0 (one-ratio) has much lower log likelihood than the site models which allow *ω* to vary among sites, indicating highly variable selective pressure along the protein. The M1a-M2a test gave 2Δℓ = 1.3, and the difference was not significant. While the parameter estimates under M2a suggested a small proportion of sites with *ω*>1, the BEB calculation [17] detected no sites with high posterior probability of being under positive selection (*P*<0.6 for all sites). The M7-M8 comparison is significant, with 2Δℓ = 11.32. The BEB calculation suggested three sites (897N, 914N, 919I) to be potentially under positive selection, with 0.80<*P*<0.86. Thus both models M2a and M8 provide some evidence for presence of sites under positive selection, but the disagreement between the two tests and the lower posterior probabilities for sites indicate that the evidence is not strong.

**Table 2:** Log-likelihood values and parameter estimates under site models for the nine mammalian BRCA1 genes.

Model	*p*	ℓ	Estimates of parameters	Positively selected sites
M0: one-ratio	26	−20,210.50	*ωˆ* = 0.585	None
M1a: neutral	27	−20,139.17	*pˆ*_0_ = 0.484, *pˆ*_1_ = 0.515	
			*ωˆ*_0_ = 0.225, *ω*_1_ = 1	
M2a: selection	29	−20,137.43	*pˆ*_0_ = 0.508, *pˆ*_1_ = 0.461, *pˆ*_2_ = 0.031	644M 897N 914N 919I (BEB, 0.5<*P*<0.6)
			*ωˆ*_0_ = 0.250, *ω*_1_ = 1, *ωˆ*_2_ = 2.276	
M7: beta	27	−20,142.53	*pˆ* = 0.581, *qˆ* = 0.365	Not allowed
M8: beta&*ω*	29	−20,136.87	*pˆ*_0_ = 0.882, *pˆ* = 1.069, *qˆ* = 1.003	897N, 914N, 919I (BEB, 0.80<*P*<0.86)
			*pˆ*_1_ = 0.118, *ωˆ* = 1.706	

Note.— *p*: Number of parameters including 15 branch lengths in the tree (Figure 4). Estimates of *κ* range from 3.4 to 3.6 among models. Sites potentially under positive selection are listed for different models, with the human sequence used as reference. The posterior probability *P* that a site is from the positive-selection class is calculated using BEB [17].

A plausible explanation for the conflicts between the joint analysis and the pairwise tests is that the selective pressure on the protein has been variable among lineages, and the various tests used here either average over sites or average across lineages, leading to somewhat inconsistent results. Previously Huttley *et al.* [2] detected positive selection in BRCA1 affecting the human and chimpanzee lineages. Indeed estimates from the free-ratios model, which assigns an *ω* ratio to every branch on the tree [18], suggested that the human and chimpanzee branches had the highest average *ω* ratios.

## Discussion

A search in the literature reveals that sliding-window analysis is widely used in molecular evolution, population genetics, and comparative genomics. In between-species comparisons, it has been used to detect regions of protein under selective constraint [19] and to assess local variations in certain properties of a protein such as solvent accessibility [20] and amino acid hydrophobicity [21]. In population genetics, it has been used to identify variations in synonymous and nonsynonymous polymorphisms within species [22]–[28], to detect balanced selection [29], to detect recombination in a gene sequence [30], [31], and to detect associations between SNPs and human diseases [32]. We do not claim that all those analyses are invalid. Indeed, Andolfatto *et al.* [33] corrected for multiple tests when they used a sliding window analysis to detect recombination. Tajima [34] discussed determination of the optimal window size. Furthermore, Ardell [35] wrote a program for performing neutrality test in a sliding window analysis by adjusting for multiple testing. Similarly Talbert *et al.* [36] used Comeron's [6] program K-estimator to conduct a sliding-window analysis of the gene sequences of the mammalian centromere protein C (CENP-C) to detect regions under purifying and positive selection. Comeron's sliding-window approach does not correct for multiple testing, but Talbert *et al.* used a trial-and-error approach to decide empirically that positive selection was supported only if *ω*>1.5 and purifying selection was indicated by *ω*<0.67 in sliding windows of 33 codons. The trial-and-error approach was an attempt to guide against the high false positives of the sliding-window analysis.

We suggest that if a certain trend is known to exist along the sequence, it is legitimate to use sliding windows to visually illustrate it. Certain amino acid properties (such as hydrophobicity) may be expected to vary gradually along the protein sequence, because neighboring residues are often in the same secondary structural categories or in the same protein fold. If such a trend is not known to exist, it is in general invalid to use sliding windows to infer the trend, because the approach will always generate a trend whether or not one exists. In addition, one has to correct for multiple testing if sliding windows are used to detect significant departures in a certain property of the molecule from null or neutral expectations. Many studies, both early and recent, did not use sliding-window analysis appropriately due to lack of an *a priori* hypothesis to stipulate the existence of the trend and due to lack of correction for multiple testing.

## Materials and Methods

### Mammalian BRCA1 genes

We retrieved from GenBank sequences for the breast-cancer gene BRCA1 from nine mammalian species: human (NM_007294), chimpanzee (AY365046), gorilla (AY5890), orangutan (AY589040), macaque (AY58904), cow (NM_178573), dog (U50709), mouse (U35641) and rat (AF036760). The sequences were aligned manually. Codons with alignment gaps were removed from all species, with 1768 codons in every sequence.

### Sliding-window analysis

The data in each sliding window were analyzed using the codeml program in the paml package [13] to fit codon model M0 (one ratio). This model involves the following parameters: sequence divergence *t*, measured in the number of nucleotide substitutions per codon, the transition/transversion rate ratio *κ* and the rate ratio *ω* = *d_N_*/*d_S_*. Codon frequencies were estimated using the observed frequencies (the Fcodon model), while other parameters were estimated by ML.

### Likelihood ratio test under site models

Two likelihood ratio tests of positive selection were implemented using the codeml program [13], [37], [38]. The first test compares M1a (neutral) against M2a (selection). M1a assumes two site classes with 0≤*ω*_0_<1 (conserved sites) and *ω*_1_ = 1 (neutral sites), while M2a (selection) adds an extra class with *ω*_2_≥1. The second test compares M7 (beta) against M8 (beta&*ω*). M7 assumes a beta distribution beta(*p*, *q*), while M8 adds an extra site class with *ω_s_*≥1. In both tests, twice the log likelihood difference was compared against 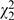 [13].

### Simulation to evaluate the false positive rate of sliding-window analysis

Data sets consisting of a pair of sequences were simulated under a codon model of neutral evolution and analyzed using sliding windows to test for positive selection. Two null models were assumed to simulate datasets, with the number of replicates to be 1000. The first model was M0 (one-ratio) with *ω* = 1. The second was M1a (neutral) with *p*_0_ = *p*_1_ = ½, *ω*_0_ = 0 and *ω*_1_ = 1. In both models, the sequence distance was fixed at *t* = 1 nucleotide substitution per codon, and the transition/transversion rate ratio was fixed at *κ* = 1. Codon frequencies were assumed to be equal (1/61). Each simulated data set was analyzed using sliding windows, with an LRT used to test whether the single *ω* in M0 (one-ratio) is significantly greater than 1. The null distribution is the 50∶50 mixture of point mass 0 and 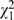 [39], with the critical value to be 2.71 at the 5% level. Positive selection was claimed to be detected for the replicate data set if the LRT was significant in at least one window. The sequence length used was 300 codons.
