# Correction: The Trouble with Sliding Windows and the Selective Pressure in BRCA1

**DOI:** 10.1371/annotation/5b41243b-64ba-47ab-bcb7-d45817547a95

**Published:** 2008-12-05

**Authors:** Karl Schmid, Ziheng Yang

The second affiliation appears incorrectly. It should read: Department of Biology, University College London, London, United Kingdom

